# *LTBSG1*, a New Allele of *BRD2*, Regulates Panicle and Grain Development in Rice by Brassinosteroid Biosynthetic Pathway

**DOI:** 10.3390/genes9060292

**Published:** 2018-06-11

**Authors:** Ran Qin, Dongdong Zeng, Chengcong Yang, Delara Akhter, Md. Alamin, Xiaoli Jin, Chunhai Shi

**Affiliations:** 1 Department of Agronomy, Zhejiang University, Hangzhou 310058, China; ranqin89@zju.edu.cn (R.Q.); njdy9791@hotmail.com (D.Z.); chengcongyang@zju.edu.cn (C.Y.); 11516090@zju.edu.cn (D.A.); alamin@zju.edu.cn (M.A.); jinxl@zju.edu.cn (X.J.); 2 Department of Genetics and Plant Breeding, Sylhet Agricultural University, Sylhet 3100, Bangladesh

**Keywords:** rice, *LTBSG1*, longer top branch, shorter grain, brassinosteroid (BR)

## Abstract

Panicle architecture and grain size are two important agronomic traits which determine grain yield directly in rice. In the present study, a mutant named *ltbsg1* (*longer top branch and shorter grain 1*) was isolated from the cultivar “Zhenong 34” (*Oryza sativa* L. ssp. *indica*) by ethyl methane sulfonate (EMS) mutagenesis. The target gene was studied through phenotype observation, genetic analysis, map-based cloning and functional analysis. The histocytological analysis indicated that the elongated top branch and shorter grain of mutant *ltbsg1* were caused from the defects of cell elongation. The *ltbsg1* gene in mutant revealed a single nucleotide substitution (G-A) in the exon 2 of *LOC_Os10g25780*, causing an amino acid variation (Glycine-Arginine) in the FAD (Flavin-adenine dinucleotide)-binding domain of delta (24)-sterol reductase, which was involved in the brassinosteroid (BR) biosynthesis. *LTBSG1* was constitutively expressed and the protein was widely localized in chloroplast, nucleus and cytomembrane. The *ltbsg1* seedlings had a lower endogenous BR level and could be restored to the phenotype of wild type by exogenous BR. The *LTBSG1* knock-out lines showed similar phenotype defects as mutant *ltbsg1*, which confirmed that *LTBSG1* was responsible for top branch elongation and grain size reduction. Furthermore, *LTBSG1* along with other BR-related genes were feedback-regulated due to their obvious altered expression in mutant *ltbsg1*. This study demonstrated that *LTBSG1* could play a new role in regulating panicle and grain development by BR biosynthetic pathway.

## 1. Introduction

Rice (*Oryza sativa* L.) is one of the most important cereal crops, which feeds over half of the world’s population. With the rapid increase of population, the world is facing more serious food security challenges. The improving of crop productivity is, hence, becoming ever more urgent and important in the rice breeding. The panicle number per plant, the grain number per panicle and the grain weight were the three main factors which determines the rice grain yield directly [1]. The panicle number per plant is largely influenced by the tillering ability; the grain number per panicle is closely related to the panicle length, the branch number, the branch length, the grain density and fertility; while the grain weight is mainly dependent on the size of grain referring to a combination of the grain length, the grain width and the thickness [2,3]. Thus, both panicle architecture and grain size are important agronomic traits directly affecting the grain yield, which have been the ongoing major purposes of crop improvement programs. In recent decades, the panicle architecture genes including *FZP*, *DEP1*, *LP*, *FUWA* and the grain size genes including *GW2*, *GL3*.*1*, *TGW6*, *GL7*, *qSW5*/*GW5* and *GW7* have been characterized consecutively [4,5,6,7,8,9,10,11,12,13,14]. However, the molecular mechanisms underlying the regulations of panicle architecture and grain size remained unclear, especially for the genes controlling the panicle morphology and grain size.

The existing data suggested that the panicle architecture and the grain size were not only controlled by genetic factors, which determined the states of inflorescence and spikelet meristem and regulated the cell division but also affected by the external environment (temperature, light, humidity) and hormone signals such as auxin (IAA), cytokinin (CK), gibberellin (GA) and brassinosteroids (BRs). BRs, a class of plant steroidal hormones, were involved in multiple development progresses in plants, such as organ elongation, vascular differentiation, light signaling, seed germination, reproductive development, biotic and abiotic stress response [15,16,17]. Brassinolide (BL) was the most bioactive compound among the BRs. BR biosynthesis and signaling pathways are complex regulatory processes involving multiple genes. Utilizing many mutants, the BR pathway has been transparently studied in dicotyledons *Arabidopsis*. BRs are perceived by a plasma membrane-localized BRI1 (BR INSENSITIVE 1)-BAK1 (BRI1-ASSOCIATED RECEPTOR KINASE 1) receptor complex and terminate in the nucleus [18,19,20,21], then a phosphorylation-dephosphorylation cascade involving the GSK3 (GLYCOGEN SYNTHASE KINASE 3)-like kinase BIN2 (BR INSENSITIVE 1), two downstream plant-specific transcription factors BRZ1 (BRASSINAZOLE-RESISTANT 1) and BES1 (BRI1-EMS-SUPPRESSOR 1) [17,22,23,24]. The dephosphorylated forms of BES1 and BZR1 in the nucleus could bind to the promoter of thousands of target genes and finally activate or repress their transcription [25,26]. However, the BR pathway in rice, a model plant of monocots, was less understood. Until now, there are only some BR related genes that have been identified from previous studies, which included BR biosynthetic genes *BRD1*, *D2*, *BRD2*, *D11*, *CPD1*, *CPD2* and *OsDWARF4* [27,28,29,30,31,32], as well as BR signaling gene *D61*/*OsBRI1*, *OsBZR1*, *14-3-3*, *BU1*, *OsBAK1* and *DLT* [33,34,35,36,37].

In most cases, BR-deficient or -insensitive mutants of rice showed dwarf phenotype, tortuous leaf blades or erect leaf, delayed flowering, reduced fertility and grain length [27,28,29,30,33,35]. In many studies, dwarfism and erect leaf were the most common phenotypes among all of the BR-related mutants because of the inhibitions of cell proliferation and cell elongation, which indicated that BR could regulate the organ size by affecting the cell cycle [38,39,40]. On the other hand, the studies conducted on the panicle and the grain development mediated by BR were not so much, and the genetic and molecular mechanisms remained unclear. The genes *BRD1*, *D2* and *D61* were directly acted on the BR biosynthesis and the signaling pathways, and their loss-of-function mutants were identified as the dwarf mutants with the phenotypes of short panicle and grain, but the genetic and regulated mechanism of panicle development mediated by these genes was not studied further [27,28,33]. In addition, some genes such as *XIAO*, *SG1*, *SMG1* and *GL2* controlling the grain size by cell proliferation and elongation were reported in influencing the BR responses and were speculated in involving the signaling and homeostasis of BR [41,42,43,44]. Therefore, the mechanism of BR regulating the panicle and the grain development needs a more in-depth research. Recently, a mutant *cpb1* (*clustered primary branch 1*) was identified as a new allele of *DWARF11* (*D11*), which encoded a cytochrome P450 protein and regulated the panicle architecture and grain size by BR biosynthesis pathway. In addition, using the panicle-specific promoter to drive *CPB1*/*D11* could increase the grain size and the yield in rice [30,45]. As mentioned above, much evidence has revealed that the proper genetic manipulation of BR related genes involved in the biosynthesis and signaling pathways could remarkably enhance the grain yield in plant [46,47].

Here, a BR-deficient mutant *ltbsg1* showing longer top branch and shorter grain was identified in present study. The target gene *LTBSG1* was located on chromosome 10 finally. The sequencing result showed that a nucleotide substitution (G-A) occurred at the exon 2 of *LOC_Os10g25780*, which caused the amino acid variation (Glycine-Arginine) in the FAD-binding domain where was comparatively conserved among different plants. The *LTBSG1* knock-out lines also showed similar defective phenotypes as mutant *ltbsg1*, which confirmed that *LTBSG1* was responsible for top branch elongation and grain size reduction. *LTBSG1* was a new allele of *BRD2* which encoded a delta (24)-sterol reductase and could catalyze the conversion of 24-methylenecholesterol (24-MC) to campesterol (CR) in the early stage of BR biosynthesis [29]. The panicle architecture and the grain size, as two important agronomic traits, are closely associated with the rice grain yield. In view of many reported BR-related genes were focused on the dwarfism or the leaf angle, and the new phenotype of top branch elongation in mutant *ltbsg1* was rare in rice mutants and even on BR-related mutants. Thus, this study will provide some new thoughts on the role of gene *LTBSG1*, which could regulate the yield traits such as panicle and grain development by BR pathway. 

## 2. Materials and Methods

### 2.1. Plant Materials

The mutant *ltbsg1* with longer top branch and shorter grain phenotype, was isolated from an *indica* cultivar Zhenong 34 through ethylmethane sulfonate (EMS) treatment. After multiple generations of continuous self-crossing, this mutated phenotype could be stably inherited. All the plant materials were grown in the paddy field of Zhejiang University in Hangzhou (120°19′ E, 30°26′ N), China. 

### 2.2. Measurement of Agronomic Traits

The main panicles of ten plants at mature stage were randomly selected for measuring the agronomic traits of WT and *ltbsg1* including the plant height (cm), the branch length (cm), the branch number (No.) and the seed setting rate (%). After air-dried, ten fully developed grains were measured for the grain length (mm) and the width (mm) by using the vernier caliper. The 1000-grain weight (g) was estimated from the weight of 200 grains and repeated three times.

### 2.3. Histocytological Analysis

To analyze the paraffin section, the fresh samples were fixed in FAA (3.7% formaldehyde, 50% ethanol and 5.0% glacial acetic acid) overnight at 4 °C, then dehydrated using a graded ethanol series (50%, 70%, 85% and 95% for 90 min each and 100% for 60 min) and finally embedded in paraffin. The embedded tissue sections (10 μm) were de-paraffined with xylene, and then stained with safranin and fast green. Finally, digital images were collected by the light microscopy (LEICA DMI4000, Wetzlar, Germany).

For scanning electron microscopy (SEM), fresh samples were fixed in 2.5% glutaraldehyde solution for at least 2 h, and then washed with a sodium phosphate buffer (0.1 M, pH 7.2) three times. The samples were fixed in 1% osmic acid for 1.5 h, and then dehydrated through an ethanol series. After incubated in the ethanol-isoamyl acetate (1:1, *v*/*v*) and isoamyl acetate, in turn, the samples were dried, mounted and coated with gold. Finally, they were observed and photographed by the scanning electronic microscope (HITACHI TM-1000, Tokyo, Japan).

### 2.4. BR Sensitivity Test

The BR sensitivity test of the seedlings and the lamina joint (between leaf sheath and blade) were performed as described previously [40]. To detect the BR sensitivity of mutant *ltbsg1*, 24-EBL (24-Epibrassinolide) (Sigma Aldrich, Saint Louis, MO, USA), a type of active BR, was selected in this study. Firstly, a 0.010 M mother solution was made by dissolving 25 mg 24-EBL solid powder in 2.5 mL of 95% (*v*/*v*) ethanol completely. In addition, then we added this mother solution of 0 μL, 0.1 μL, 1 μL, 10 μL and 100 μL into liquid culture solution (1 L) to prepare the test solution of 0.000 µM, 0.001 µM, 0.010 µM, 0.100 µM and 1.000 µM 24-EBL, respectively. The sterilized seeds of WT and mutant *ltbsg1* were grown in the liquid culture solution with different doses of 24-EBL in a constant growth chamber (28 °C, 16 h/light and 8 h/dark). Then the seedlings at the three-leaf stage were photographed. In the lamina joint bending assay, the detached lamina joints were photographed after incubation for 7 days under the dark condition.

### 2.5. The Determination of Endogenous Brassinolide

Whole plants of WT and mutant *ltbsg1* without any BR treatment were harvested at the 8th week after germination for measuring the endogenous brassinolide level. The methods of extraction and quantification of brassinolide were in reference to that described by Huo et al. [48]. The brassinolide, a standard in this experiment, was purchased from Sigma Aldrich with a purity of 90%. 

The fresh samples were grinded to powder in liquid nitrogen with a mortar and pestle. 2 g powder was extracted in 10 mL of 80% methanol (precooled at 4 °C) for 2 h at 4 °C. Then centrifugation at 10,000 r/min, 4 °C for 5 min, and the supernatant was transferred to the Bond Elut prepacked column (Aglient, Santa Clara, CA, USA) and eluted with 3 mL of methanol. Using methanol diluted the eluent obtained in the last step by 50% and transferred it to the strata-X cartridge (33 mm, 3 mL) (Phenomenex), eluting with 3 mL of methanol. After dried by the pressure blowing concentrator, the dry matter was dissolved with 200 μL of methanol. The solution was filtered with a 0.22 μm filter, and it stored at −20 °C until HPLC-MS/MS (High performance liquid chromatography-tandem mass spectrometry) analysis.

The HPLC separation was performed based on the system of Aglient1290 High Performance Liquid Chromatography (Aglient, Santa Clara, CA, USA), and the analytical column was a ZORBAX SB-C18 Reversed-Phase Column (2.1 × 150, 3.5 μm). The flow rate of mobile phase was set at 0.35 mL/min with column temperature of 35 °C and injection volume of 5 μL. The separation was performed by gradient elution using mobile phase (A) (0.1% formic acid) and the aqueous organic phase (B) (Methanol). The gradient elution program was employed during the separation process (Solution A: 80% in 0–2 min, 80–95% in 2–3.5 min, 95% in 3.5–6 min, 95–80% in 6–6.1 min and 80% in 6.1–10 min). The HPLC system was coupled to a SCIEX-6500Q trap (MS/MS), (SCIEX, Redwood City, CA, USA) in the positive mode. Data was acquired by the multiple reaction monitoring (MRM) mode. The capillary voltage was set at 5500 V and the rate of nebulization gas was set as 800 L/h at 550 °C. The ion source temperature was set at 60 °C. The protonated molecule was chosen as the precursor ion and the most intensive product ion was selected for the quantification. The selected quantification ion plus another specific product ion were chosen for the confirmation. MRM mass spectrometric parameters of the two analytes were summarized in Table 1.

### 2.6. Genetic Analysis and Map-Based Cloning of ltbsg1

The segregation ratio of *ltbsg1* phenotype and WT plants in the F_2_ population from the crossing *ltbsg1* and Zhenong 34 plants was analyzed by the Microsoft Excel.

To map the gene *ltbsg1*, F_2_ population was constructed by the crossing from *ltbsg1* with Zhenongda 104 (*Oryza sativa* L. ssp. *japonica*) plants. Totally 729 individuals with clear *ltbsg1* phenotype from the F_2_ population were selected for the gene mapping by using the mapping method performed as described by Zhang et al. [49]. The polymorphic primers were designed by DNASTAR and Primer premier 5.0 software based on the sequence differences between *indica* (*Oryza sativa* L.) and *japonica* (*Oryza sativa* L.). To investigate the functional annotations of genes in the candidate region, databases of RGAP (Rice Genome Annotation Project, http://rice.plantbiology.msu.edu/cgi-bin/gbrowse/rice/) and RiceData (China Rice Data Center, http://www.ricedata.cn/gene/) were used, thereby sequencing the candidate genes of WT and *ltbsg1* to confirm the mutation site. The gene structure and sequence were referred to http://ensembl.gramene.org/Oryza_indica/Info/Index. The primes for the gene mapping are listed in Table S1A.

### 2.7. Generation of Knock-Out Transgenic Plants

To construct the knock-out vector of *LTBSG1*, the pYLCRISPR/Cas9P_ubi_-H system was used in this study [50]. The sequence ATGGATTCTGGTGATCTTTG close to the start codon of *LTBSG1* was selected as the target of single guide RNA (sgRNA). The overlapping PCR was used to introduce the target sequences into sgRNA expression cassettes, and then the sgRNA expression cassette was cloned into the pYLCRISPR/Cas9P_ubi_-H binary vector by golden gate cloning strategy. Finally, the vector was introduced into Nipponbare (*Oryza sativa* L. ssp. *japonica*) callus by *Agrobacterium*-mediated transformation using EHA105 strain. To identify mutation by CRISPR (Clustered regularly interspaced short palindromic repeats)/Cas9 (CRISPR-associated 9) editing, the primers flanking the designed target site of genomic DNA from transgenic plants were used for the PCR amplification and sequencing. Primers used for vector construction are listed in Table S1B. In this experiment, totally eight positive lines by CRISPR/Cas9 editing were obtained. Three homozygous lines with different mutations (*Cas-k1*, *Cas-k2* and *Cas-k3*) and Nipponbare were used for analysis. The data for expression level of *LTBSG1* in each knock-out line was derived from three biological replications. Ten plants from each line were selected for the measurements of agronomic traits and the details followed the way of the WT and mutant *ltbsg1*.

### 2.8. β-Glucuronidase Assay

β-glucuronidase (GUS) assay in transgenic plants was performed as previously described by Jefferson et al. [51]. To generate the promoter: GUS vector, a 2262-bp fragment upstream from the ATG codon of *LTBSG1* was amplified. Primers used for vector construct are listed in Table S1B. After the amplified fragment ligated into the binary vector pCAMBIA1301 upstream of the GUS reporter gene, this vector was introduced into Nipponbare callus by Agrobacterium-mediated transformation using EHA105 strain. The tested tissues were immersed in a solution (1 mM 5-bromo-4-chloro 3-indolyl-glucuronic acid, 100 mM sodium phosphate (pH 7.0), 0.1 mM EDTA, 0.5 mM ferricyanide, 0.5 mM ferrocyanide and 0.1% Triton X-100). Then the samples were vacuumed for half an hour and incubated at 37 °C, for 24 h. After removal chlorophyll from by 70% ethanol, digital images were taken by light microscopy (LEICA DMI4000, Wetzlar, Germany).

### 2.9. Subcellular Localization of LTBSG1

For subcellular localization, the full-length coding sequence of *LTBSG1* without the stop codon was amplified (Primers are listed in Table S1B) and ligated into the empty GFP (Green Fluorescent Protein) vector. Then the empty vector and the fusion constructs GFP-LTBSG1 vectors were introduced into the rice protoplasts as described by He et al. [52]. The fluorescence was detected by laser confocal microscopy (ZEISS LSM 700, Jena, Germany).

### 2.10. RNA Isolation and qRT-PCR Analysis

Total RNA was extracted using Trizol reagent as the manufacturer’s protocol (Invitrogen, Carlsbad, CA, USA). PrimeScript RT reagent Kit with gDNA Eraser and SYBR Premix Ex Taq II were used for qRT-PCR (Quantitative Real Time-PCR) as described by instruction of Takara Company (Tokyo, Japan). Primers for qRT-PCR were listed in the Table S1C. The expression levels were analyzed using a Real-Time System of Roche LightCycler^®^ 96 (Basel, Switzerland) with rice *OsActin* as an internal control. The reaction solution (20 μL) contained 0.8 μL of each primer (10 μM), 10 μL 2 × SYBR Premix Ex Taq II, 1.6 μL cDNA and 6.8 μL ddH_2_O. The three-step protocol was performed in this qRT-PCR: activation at 95 °C for 30 s, followed by 40 cycles of denaturation at 95 °C for 5 s, annealing at 55 °C for 20 s, and extending at 72 °C for 10 s. Values of expression levels represent the means ± standard deviation (SD) of three biological replicates (*n* = 3). * *p* < 0.05; ** *p* < 0.01.

### 2.11. Alignment Analysis and Phylogenic Analysis

All the protein sequences were downloaded from the NCBI (http://www.ncbi.nlm.nih.gov/BLAST/), using the LTBSG1 full-length protein sequence as a query against the nonredundant protein database. Amino acid sequence alignment was performed by the ClustalW [53]. The phylogenetic tree was constructed using the MEGA 6.0 [54] with the neighbor-joining method by 1000 bootstrap replicates.

## 3. Results

### 3.1. Phenotype Characterization of Mutant ltbsg1

**Figure 1:**
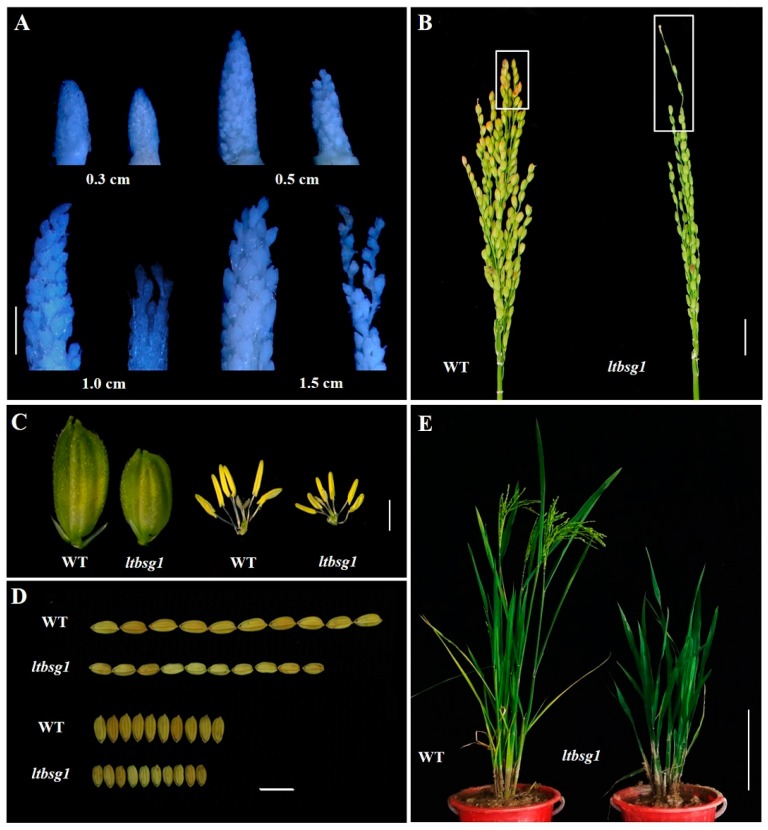
Phenotype characterization of wild type (WT) and mutant *ltbsg1* (*longer top branch and shorter grain 1*). (**A**) The young panicles of different length in WT and mutant *ltbsg1*. Bar = 2 mm. Left: WT; Right: *ltbsg1*; (**B**) The panicles of WT and mutant *ltbsg1* at grain-filling stage. The white box indicated the top branch of panicle. Bar = 2 cm; (**C**) The spikelets and flowers of WT and mutant *ltbsg1*. Bar = 2 mm; (**D**) The grains length and width of WT and mutant *ltbsg1*. Bar = 1 cm; (**E**) Plants of WT and mutant *ltbsg1* at grain-filling stage. Bar = 20 cm.

The phenotype observation showed that mutant *ltbsg1* displayed the new phenotype of longer top branch in panicle and shorter grain (Figure 1A–D; Table 2). In addition, *ltbsg1* exhibited severe dwarfism, erect lamina joint and late mature phenotype (Figure 1E; Figure S1; Table 2). In the early stage of panicle development, the panicle of *ltbsg1* was a little smaller compared with WT. When the panicle length of *ltbsg1* reached to 0.5 cm, the top branch of *ltbsg1* started to protract and it was dramatically elongated later compared with that of WT (Figure 1A). At grain-filling stage, the top branch of *ltbsg1* was much longer than that of WT, whereas the other branches were relatively short. The primary and secondary branches of *ltbsg1* were much less than those of WT (Figure 1B; Table 2). Furthermore, the spikelet and floret of *ltbsg1* were obviously smaller than those of WT (Figure 1C). The mature grain of mutant *ltbsg1* also displayed significantly reduced length and width, which led to a much decreased 1000-grain weight (16.63 g) (Figure 1D; Table 2). The pollen vitality of *ltbsg1* was much lower than that of WT, while the seed setting rate was just 8.42% correspondingly (Figure S2; Table 2).

### 3.2. LTBSG1 Affected the Top Branch and the Grain Size by Regulating the Cell Elongation

**Figure 2:**
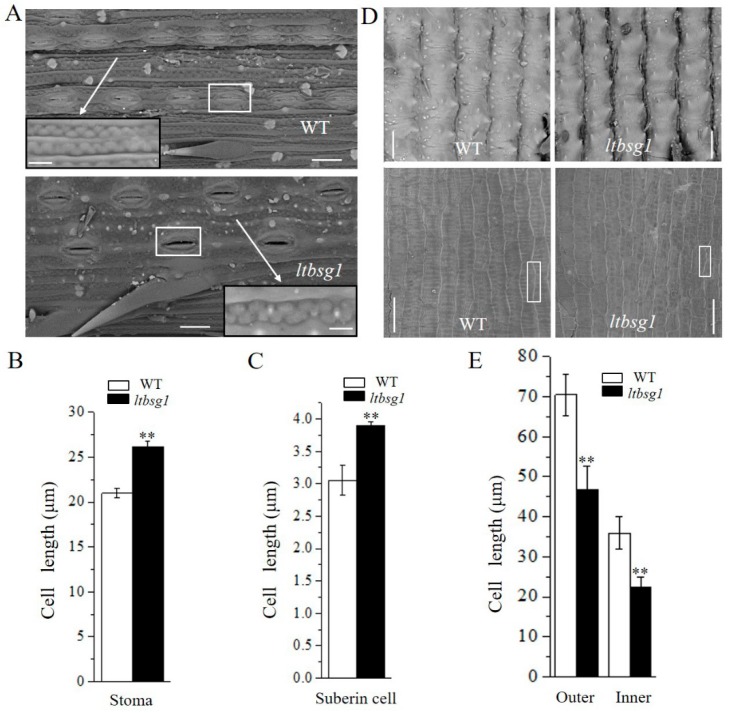
The effect of *LTBSG1* on top branch and grain development. (**A**) The scanning electron microscopic analysis of the top branch surfaces of WT and mutant *ltbsg1*. Bars = 20 μm. The white frames indicated the stoma and the white arrows indicated the magnified views of suberin cell. Bars = 5 μm; (**B**) The length of stoma in top branch surfaces of WT and mutant *ltbsg1*; (**C**) The length of suberin cell in top branch surfaces of WT and mutant *ltbsg1*; (**D**) The scanning electron microscopic analysis of spikelet hulls of WT and mutant *ltbsg1*. The outer surface and inner surface were showed up panel (Bars = 50 μm) and bottom panel (Bars = 30 μm), respectively; (**E**) The cell length of outer and inner surfaces of spikelet hulls in WT and mutant *ltbsg1*. Values represent the means ± SD (*n* = 10). ** *p* < 0.01.

To clarify whether cell proliferation or cell elongation affecting the lengths of branch and grain in *ltbsg1*, assays of scanning electron microscopes were performed in the present experiment. The lengths of stomata and suberin cell of mutant *ltbsg1* were significantly longer in top branch than those of WT, indicating that the cell elongation was exacerbated in *ltbsg1* (Figure 2A–C). However, the cell lengths of both outer and inner epidermal surfaces of spikelet were much shorter in mutant *ltbsg1* compared to those in WT, which conferred the reduced grain length of mutant (Figure 2D,E).

### 3.3. Map-Based Cloning of the ltbsg1 Gene

The genetic analysis showed that the panicle morphology of F_1_ plants was normal as that of wild type, and it produced a phenotypic separation between wild type (normal panicle) and *ltbsg1* phenotype (deformed panicle with longer top branch and shorter grain) in the F_2_ population (*ltbsg1*/Zhenong 34). In addition, the segregation ratio of wild type to *ltbsg1* phenotype accorded with a Mendel model of 3:1 ($\chi^{2}=\;0.73<\chi_{0.05}^{2}=3.84$, *n* = 408). This result indicated that the phenotype of longer top branch and shorter grain in *ltbsg1* was controlled by a single recessive nuclear gene.

**Figure 3:**
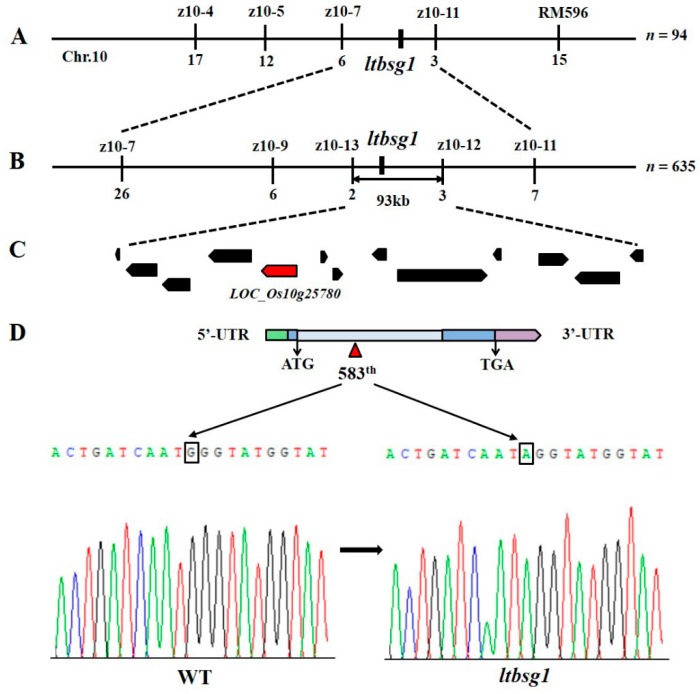
Map-based cloning and confirmation of the gene *ltbsg1*. (**A**) Primary mapping of *ltbsg1*; (**B**) The fine mapping of gene *ltbsg1*; (**C**) The candidate genes in the target region; (**D**) The gene structure and the mutation site confirmed by sequencing analysis of *LOC_Os10g25780* in WT and mutant *ltbsg1*. The black box indicated the single nucleotide mutation G (WT) to A (*ltbsg1*) at the 583th base in the exon 2. UTR: Untranslated Regions; Chr.: Chromosome.

**Figure 4:**
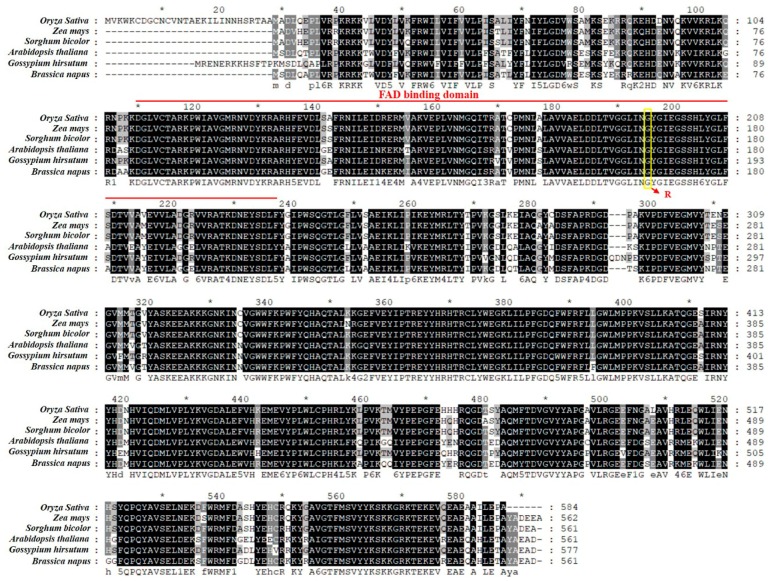
ClustalW alignment of the orthologs of LTBSG1. The orthologs of LTBSG1 from different plants were *Zea mays* (Accession Number NP_001105560.1), *Sorghum bicolor* (XP_021306085.1), *Arabidopsis thaliana* (NP_188616.1), *Gossypium hirsutum* (XP_016697581.1) and *Brassica napus* (XP_013721305.1), respectively. The red line region represented the FAD-binding domain (110-237 amino acids) and the amino acid substitution (G-R) was indicated by the yellow frame.

Map-based cloning was used for locating the *ltbsg1* gene based on the F_2_ population (*ltbsg1*/Zhenongda104). *LTBSG1* was primarily mapped on the long arm of chromosome 10 between InDel (Insertion-Deletion) markers z10-7 and z10-11 (Figure 3A). To fine-map the gene, another 635 recessive individuals and several polymorphic markers were used, and the target locus was narrowed down to a 93-kb interval between markers z10-13 and z10-12 (Figure 3B). A total of 13 putative genes were distributed in this region according to database of RGAP and the genes function annotations of them are listed in Table S2. Among these genes, one locus *LOC_Os10g25780* was annotated as FAD-linked oxidoreductase protein and it was allelic to *BRD2*, which was involved in BR biosynthesis (Figure 3C). After comparing the genomic sequences of these genes in this region between WT and *ltbsg1*, only one base substitution (G-A) at 583th base in the exon 2 of *LOC_Os10g25780* was detected, which caused an amino acid substitution Glycine-Arginine (G-R, GGG-AGG) on the FAD-binding domain (Figure 3D and Figure 4). Thus, these results suggested that *LOC_Os10g25780* was the candidate gene, which was responsible for longer top branch and shorter grain.

### 3.4. Analysis of LTBSG1 Orthologous Proteins 

The *LTBSG1* gene encoded a delta (24)-sterol reductase and played an important role in BR biosynthesis by catalyzing the conversion of 24-methylenecholesterol (24-MC) to campesterol (CR) [29]. LTBSG1 contained a FAD-binding domain. To identify the conservation of LTBSG1 protein sequences, another five orthologs of LTBSG1 from *Zea mays*, *Sorghum bicolor*, *Arabidopsis thaliana*, *Gossypium hirsutum* and *Brassica napus* were compared. They shared high similarity in the FAD-binding domain and the amino acid (G) where the substitution occurred was unified among the six plants (Figure 4). This result indicated that this domain sequence was highly conserved and played a very important role in maintaining the function of LTBSG1. As shown in phylogenetic analysis of 20 representative proteins, compared with the dicots, the LTBSG1 protein was more closely related to the monocots *Oryza sativa japonica*, *Oryza brachyantha*, *Setaria italic*, *Zea mays* and *Sorghum bicolor* (Figure S3). 

### 3.5. Subcellular Localization of LTBSG1

**Figure 5:**
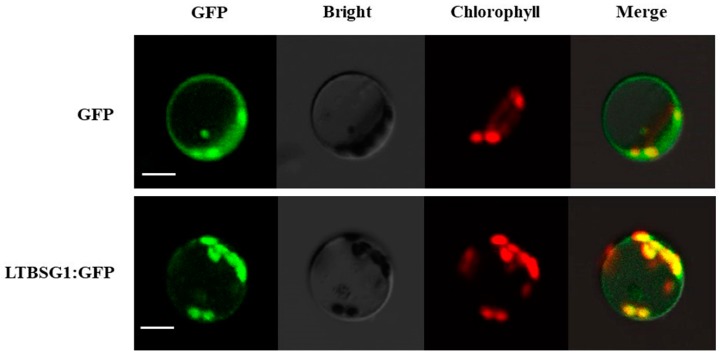
Subcellular localization of LTBSG1-GFP in rice protoplasts. Free GFP (Green Fluorescent Protein) (up panel) and LTBSG1-GFP (bottom panel) vectors were introduced into rice protoplasts. The horizontal four columns referred to GFP fluorescence images, bright images, chlorophyll spontaneous light images and their merged images for the same cells, respectively. Bars = 50 μm.

To confirm the subcellular localization of LTBSG1, the LTBSG1-GFP fusion vector was transiently expressed in the rice protoplasts. LTBSG1-GFP signals in the rice protoplasts were distributed almost in whole cell, which was strongly expressed in chloroplast and slightly in nucleus, cytomembrane (Figure 5). Therefore, the widely localization of LTBSG1 implied that it might be involved in multiple functional pathways in plant development.

### 3.6. Expression Pattern of LTBSG1

**Figure 6:**
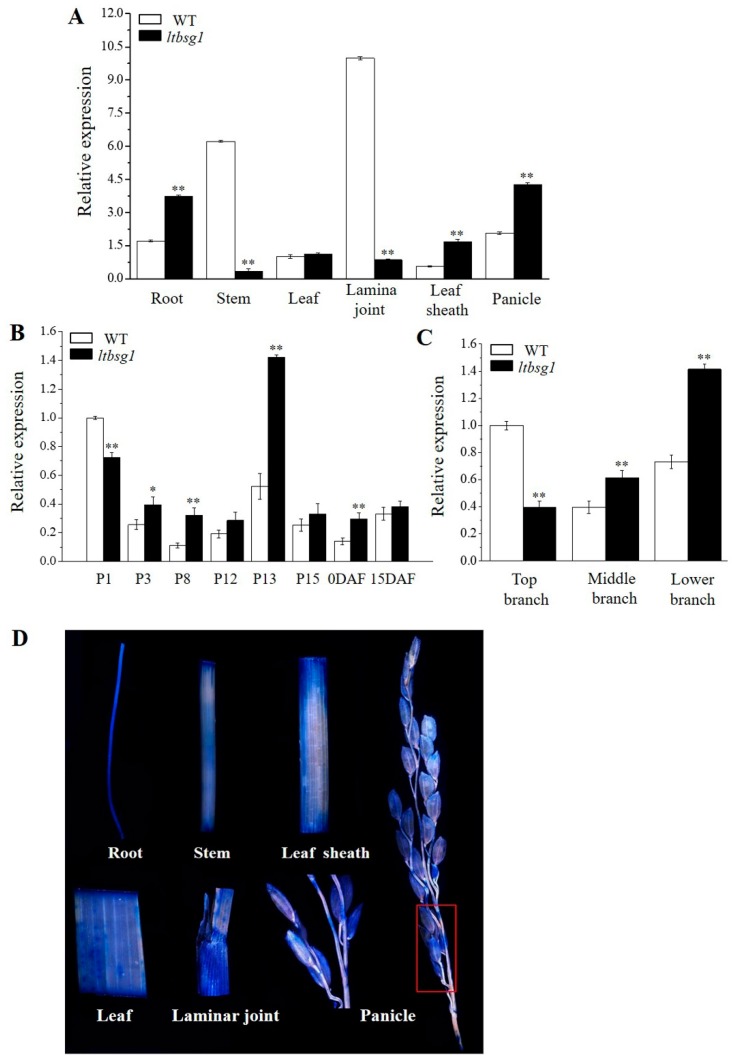
Expression pattern analysis of *LTBSG1*. (**A**) Temporal and spatial patterns of *LTBSG1* gene in WT and mutant *ltbsg1* at heading stage; (**B**) *LTBSG1* gene expression in panicles of 1 cm (P1) to 15 cm (P15), flowering day (0DAF) and 15 days after fertilization (15DAF); (**C**) The expression of *LTBSG1* in different branches of young panicle in WT and mutant *ltbsg1*; (**D**) β-glucuronidase (GUS) assay staining analysis of the *LTBSG1* promoter in different tissues. The red box in panicle was magnified. The rice *OsActin* gene was used as an internal control. Values represent the means ± SD of three biological replicates (*n* = 3). * *p* < 0.05; ** *p* < 0.01.

The expression profile of *LTBSG1* at the heading stage showed that it could be expressed in all the tested organizations and was highly expressed in root, stem, lamina joint and panicle in WT. The expression levels of *LTBSG1* in stem and lamina joint were much down-regulated in mutant *ltbsg1* and up-regulated in root, leaf sheath and panicle, but the difference in leaf was not significant between WT and mutant *ltbsg1* (Figure 6A). As the morphological states of branch and spikelet could be determined before the grain-filling stage, panicles in the length of 1 cm (P1) to 15 cm (P15), flowering day (0 DAF) and 15 days after fertilization (15 DAF) were selected for determining the expression levels of *LTBSG1*. *LTBSG1* transcripts in both WT and *ltbsg1* accumulated more in P1 and then decreased much in later several stages, and it reached the strongest level of the expression in P13 and then became lower again until the 15 DAF. Obviously, the expression levels of *LTBSG1* in mutant *ltbsg1* except for P1 were higher than those in WT during panicle development although they were not significant for P12 and P15 (Figure 6B). Moreover, the expression distinction of *LTBSG1* in different branches of one panicle was also observed. The expression level of *LTBSG1* in top branch of WT was significantly higher than those in the middle and lower branches, while that in top branch of mutant *ltbsg1* was expressed at the lowest level (Figure 6C). These results revealed the expression variance of *LTBSG1* in top branch between mutant and WT, which might be the reason for the elongation of the top branch in mutant. In agreement with the above gene expression profile in Figure 5A, LTBSG1-GUS could be expressed in all of the examined tissues at heading stage especially for root, lamina joint, spikelet and branch of the panicle (Figure 6D).

### 3.7. The Expression Analysis of Panicle Architecture and Grain Size Related Genes

**Figure 7:**
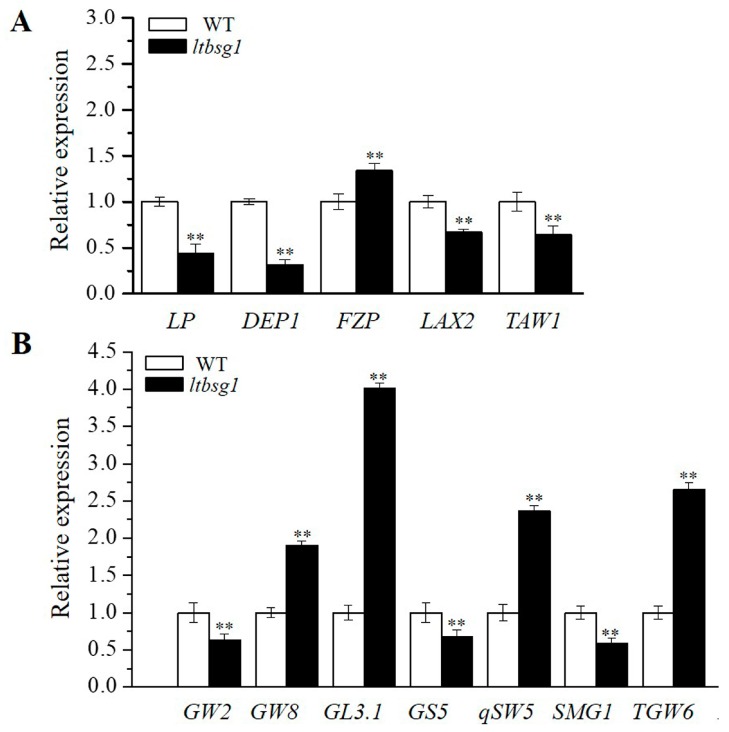
The expression of genes related to the panicle and the grain development in WT and mutant *ltbsg1*. (**A**) The expression of genes related to the panicle development in young panicles of WT and mutant *ltbsg1*; (**B**) The expression of genes related to the grain size in young panicles of WT and mutant *ltbsg1*. The rice *OsActin* gene was used as an internal control. Values represent the means ± SD of three biological replicates (*n* = 3). ** *p* < 0.01.

To identify the role of *LTBSG1* in the regulatory pathway about panicle and grain development, the expression levels of genes associated with the panicle architecture and the grain size were examined correspondingly. The results showed that the expressions of *LP*, *DEP1*, *LAX2* and *TAW1* were much down-regulated in the mutant *ltbsg1*, whereas *FZP* was higher increased compared with those in WT (Figure 7A). As well, the expression levels of genes related to the grain size revealed that the expressions of *GW8*, *GL3.1*, *qSW5* and *TGW6* but not *GW2*, *GS5* and *SMG1* were significantly increased in mutant *ltbsg1* (Figure 7B). These results suggested that *LTBSG1* might regulate the panicle and the grain development relying on the panicle and the grain regulation pathway by affecting these related genes’ expression.

### 3.8. ltbsg1 Was a BR-Deficient Mutant and LTBSG1 Was Feedback-Regulated by 24-EBL

**Figure 8:**
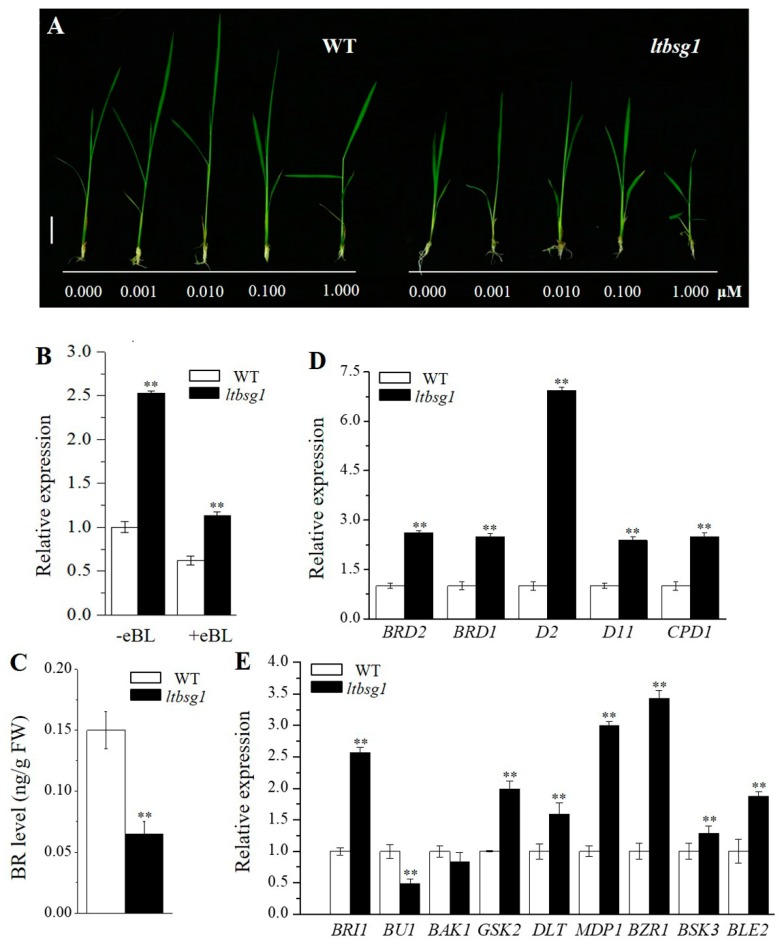
Rescue of the phenotype of mutant *ltbsg1* by 24-EBL and the feedback regulation of BR-related genes. (**A**) Phenotypic observation of WT (left) and mutant *ltbsg1* (right) at three-leaf stage treated with different doses of 24-EBL (0.000 to 1.000 μM). Bar = 1.5 cm; (**B**) The expression of *LTBSG1* in leaf treated with 0.01 μM 24-EBL; (**C**) Quantification of endogenous brassinolide contents of WT and mutant *ltbsg1*; (**D**) The expression levels of genes related to BR biosynthesis in young panicles of WT and mutant *ltbsg1*; (**E**) The expression levels of genes related to BR signaling in young panicles of WT and mutant *ltbsg1*. The rice *OsActin* gene was used as an internal control. Values represent the means ± SD of three biological replicates (*n* = 3). ** *p* < 0.01.

It is well known that the altered plant height and lamina joint bending were the typical responses to BR in rice for which were highly sensitive to BR. Those were also considered as the proofs to determine whether mutant was BR-deficient or BR-insensitive [28]. To test the BR sensitivity of mutant *ltbsg1*, different doses of 24-EBL, a bioactive BR compound, were used at the seedling stage. The mutant *ltbsg1* showed the highly response to 24-EBL-like WT in both seedling height and lamina joint bending, thus, it was considered to be a BR-deficient mutant. The heights of seedlings for both WT and were increased with the increasing 24-EBL dose and it reached the highest at 0.010 µM, then suppressed by 24-EBL of 0.100 µM and 1.000 µM gradually (Figure 8A). It indicated that the plant height was dose-dependent on BR. However, the lamina joint bending was positive promoted with the dose increasing. The bending of lamina joint in mutant *ltbsg1* at 0.000 µM 24-EBL was almost invisible, but it could bend more greatly as the 24-EBL dose increased, even the leaf angle was near 90° in both WT and mutant *ltbsg1* at 1.000 µM (Figure 8A). In terms of the response to 24-EBL, 0.010–0.100 µM might be the optimal concentration region for recovering the phenotype of mutant. Given that the expressions of BR biosynthesis genes, such as *BRD1*, *D2* and *OsDWARF4* were feedback regulated by the end product of the BR biosynthesis pathway [27,28,32], the effect of 24-EBL on *LTBSG1* expression was studied here. Without 24-EBL, the *LTBSG1* expression level was higher in mutant *ltbsg1* seedlings compared with that in WT. In addition, it was dramatically reduced in WT or mutant plants after treated with 24-EBL, implying that the expression of *LTBSG1* was feedback-regulated by 24-EBL (Figure 8B). Correspondingly, the level of endogenous brassinolide in mutant *ltbsg1* was reduced to one-half of that in WT, confirming that the mutant *ltbsg1* was deficient in the biosynthesis of BR (Figure 8C).

In addition, the expression levels of BR biosynthesis genes *BRD1*, *D2*, *D11* and *CPD1* in young panicle of mutant *ltbsg1* were much higher than those in WT (Figure 8D). The significantly higher expression of gene *D2* among these biosynthetic genes indicated it was the most responsive to BR defect. This result indicated the feedback regulations of BR biosynthetic genes in young panicle. Meanwhile, BR signaling genes including *BRI1*, *GSK2*, *DLT*, *MDP1*, *BZR1*, *BSK3* and *BLE2* except for *BU1* and *BAK1* had the increased expression levels in mutant *ltbsg1* (Figure 8E). The responses of BR signaling genes in mutant *ltbsg1* revealed that the BR signaling genes were required in the feedback regulation of BR biosynthetic genes.

### 3.9. The Confirmation of LTBSG1 Gene Function

**Figure 9:**
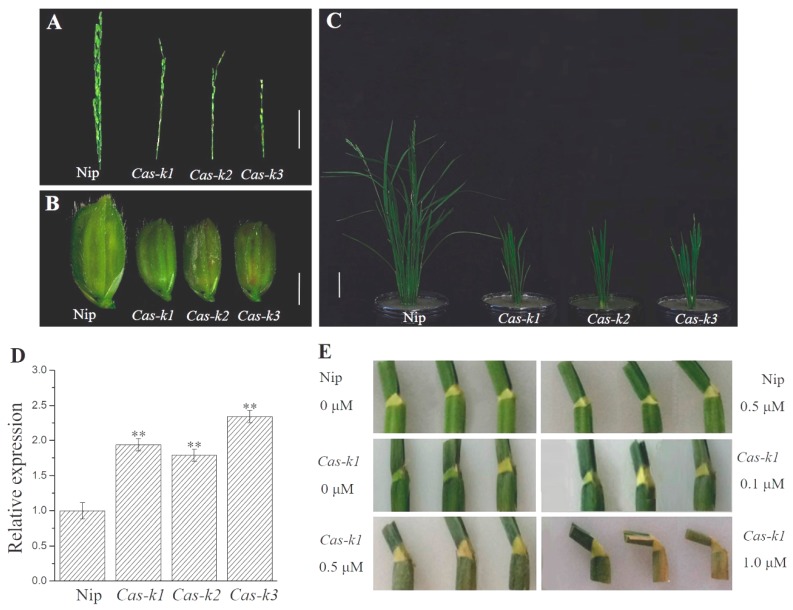
Phenotype analysis of three knock-out lines *Cas-k1*, *Cas-k2* and *Cas-k3*. (**A**) The morphological analysis of panicles of knock-out lines. Bar = 5 cm; (**B**) The morphological analysis of spikelets in knock-out lines. Bar = 2 mm; (**C**) Plant phenotype of knock-out lines. Bar = 10 cm; (**D**) The expression levels of *LTBSG1* in the young panicles of knock-out lines; (**E**) Sensitivity test of lamina joints to 24-EBL in knock-out line *cas-k1*. Values represent the means ± SD of three biological replicates (*n* = 3). ** *p* < 0.01.

Since the *indica* material Zhenong34 was difficult to regenerate in plants, Nipponbare was used for producing knock-out transgenic lines of *LTBSG1* by CRISPR/Cas9 strategy, to confirm the inactivation of *LTBSG1* contributed to the elongation of top branch and the reduction of grain size in mutant *ltbsg1*. All the eight positive lines showed similar defects on the panicle development as the mutant *ltbsg1*. Among them, three independent homozygous lines *Cas-k1*, *Cas-k2* and *Cas-k3* accompanied with nucleotide deletions at 173th bp, 174th bp and 175th bp of exon 2 respectively were selected for phenotypes analysis (Figure S4). They exhibited obvious elongation of top branches on the panicle (Figure 9A, Table S3). Meanwhile, the grains were much shorter with the decreased length and width compared to those of Nipponbare (Figure 9B, Table S3). The expression level of *LTBSG1* in young panicles of each line was much increased than that in Nipponbare, which was consistent with the expression trend in the mutant *ltbsg1* (Figure 9C). Beyond that, the plants of knock-out lines displayed severe dwarfism and the lamina joints did not show any bending and leaf blades were upright, which led to compact plant architecture in the knock-out plants (Figure 9D, Table S3). In addition, the agronomic traits of each line also showed serious defects such as the reduced grain number per panicle and seed setting rate (Table S3), which were also consistent with those of mutant *ltbsg1*. The BR sensitivity test revealed that the lamina joints of knock-out plants was hyper-sensitive to 24-EBL and it could bend more obviously with the increasing BR dose (Figure 9E). Therefore, we concluded that the gene *LTBSG1* was responsible for the longer top branch and shorter grain of mutant *ltbsg1* as well as the knock-out plants.

## 4. Discussion

The panicle architecture and grain size are important agronomic traits, which are closely related to grain production. As many mutants and genes have been identified, people are more aware of the mechanisms of panicle and grain development now. In general, rice plant showed normal panicle with the short top branch. However, the mutant *ltbsg1* in the present experiment displayed longer top branch, which was rare in previous studies. Meanwhile, the grains of mutant were much shorter than those of WT. The genetic analysis indicated that this phenotype was controlled by a single recessive gene. Through map-based cloning, the locus of *LOC_Os10g25780* in mutant *ltbsg1* revealed a single nucleotide substitution (G-A) in exon 2, causing an amino acid variation (G-R) on the FAD-binding domain. The gene *LTBSG1* was allelic to *BRD2*, which encoded delta (24)-sterol reductase functioning in the BR biosynthesis pathway by catalyzing the conversion of 24-methylenecholesterol to campesterol [29]. Interestingly, the gene *LTBSG1* was identified to be a new allele of *BRD2*, for the mutation site and phenotype were different from the previous two mutants *brd2* and *lhdd10*, even though all the three mutants showed the typical BR-deficient phenotype such as severe dwarf, erect lamina joint, late mature and low fertility [29,55]. More importantly, the new phenotype of top branch elongation in mutant *ltbsg1* was rare on BR-related mutants. Besides the longer top branch, the internode pattern among these three mutants was also obviously different. The mutant *ltbsg1* exhibited similar inhibition in internode length as *brd2*, which was in *japonica* background and had a premature stop codon on exon 2. Compared to WT, the first two internodes of *ltbsg1* from the base were so short that could not be distinguished easily, and the upper three internodes were also reduced a lot, which was caused by the inhibition of cell elongation (Figure S1). However, the allele *lhdd10* with *indica* background had a single nucleotide substitution in exon 2 and showed a very different type. The first two internodes from the base of *lhdd10* plant were almost not changed, only the upper three internodes were shortened. Given the above, the gene *LTBSG1* showing different effects on plant development might due to the different genetic background and the mutation way.

Many genes related to biosynthesis and signal transduction of BR were reported recently and had effect on plant height, panicle architecture and grain size such as *GW5*, *XIAO*, *SMG1*, *GL2*, *CPB1*/*D11* and *GS6*/*DLT*, which also influenced the BR responses and the expression of BR-related genes [13,41,42,43,44,45,56]. Previous studies suggested that BR regulated the organ size by cell elongation and cell division in rice [27,41,57]. The organs of BR-related mutants were usually short and small, but the regulation mechanism was still not clear. Here, mutant *ltbsg1* exhibited the defects in cell elongation in different organ including short stem and grain. On the other hand, it showed longer top branch due to the obvious cell elongation were (Figure 2; Figure S1). This contrary phenomenon was also occurred on previous BR related mutants *d2* and *d61* [28,33]. They not only showed the different elongation of internodes such as mutant *ltbsg1*, but also produced the over-elongation of panicle neck internode. The gene *BRI1*/*D61* was expressed higher in the uppermost and fourth internodes, and it allowed these internodes to respond to BR by inducing elongation. The expression levels of *LTBSG1* were also varied significantly among different organs, suggesting that their response to BR was also different. This might be the reason for different elongation patterns in mutant *ltbsg1*. 

A recent study suggested that the BRs could promote the pollen and grain development in rice by enhancing the expression of *Carbon Starved Anther* (*CSA*), which could directly trigger the expression of sugar partitioning and metabolic genes [58]. OsSPL16 (GW8), an SBP-domain transcription factor related to the grain width, could directly bound to the *GW7* promoter and repressed its expression to further regulate the grain size [14]. In the present study, the gene *LTBSG1* might act on the panicle and grain development relying on the related genes regulatory network. In support of this suggestion, the expression levels of most panicle and grain related genes were alerted in mutant *ltbsg1* (Figure 7). The expression level of *DEP1* was repressed in mutant *ltbsg1*, which regulated the panicle size negatively, indicating its negative relationship with *LTBSG1*. The increased expression levels of *qSW5* and *TGW6* (two negative regulators of grain size) and the decreased expression levels of *GS5* and *SMG1* (two positive regulators of grain size) in mutant *ltbsg1* might indicate that these genes also contributed to the shorter grain of mutant *ltbsg1* [10,12,43,59].

The mutants defective in BR biosynthesis pathway were usually sensitive to exogenous BR, and the defects could be rescued when given exogenous BR [29,30]. However, the mutants involved in BR signaling transduction could not perceive exogenous BR and the signal would not be passed on normally [33,35,37]. The sensitivity test to exogenous BR was also considered as the basis to determine whether it was a BR-deficient mutant or not. As mutant *brd2*, *ltbsg1* was a BR-deficient mutant given that it was highly sensitive to 24-EBL and contained less endogenous brassinolide, especially the phenotype of mutant *ltbsg1* could be recovered by a moderate dose of 24-EBL (Figure 8A,C). It was common that the biosynthesis of plant hormones was controlled by the level of the end products in a feedback manner through regulating the biosynthetic genes. Such feedback mechanism could maintain the endogenous hormone homeostasis. The phenomenon has been demonstrated on BR and other hormones, such as ethylene and GA [60,61,62]. Previous studies have revealed that expressions of genes *BRD1*, *D2* and *D11*, which encoded key enzymes involved in BR biosynthesis, were feedback regulated by bioactive BR [27,28,30]. Here, *LTBSG1* showed a similar feedback manner to the above genes, and it was negatively regulated by BR level. *LTBSG1* had a higher expression in the BR-deficient mutant *ltbsg1* than that of WT, and its expression was down-regulated after treated with 24-EBL (Figure 8B). Moreover, the other BR biosynthetic genes tested in this study such as *BRD1*, *D2*, *D11* and *CPD1* were found to have up-regulated expressions in the mutant *ltbsg1* (Figure 8D), which were consistent with the feedback regulation of these genes in BR-deficient mutants. The significantly higher expression of gene *D2* among these biosynthetic genes indicated it was the most responsive to BR defect to maintain the BR homeostasis. This feedback regulation was also found in *Arabidopsis* cytochrome P450 genes *DWF4*/*CYP90B1*, *CPD*/*CYP90A1*, *DWARF*/*CYP85A1* and *CYP90D1*, which were downregulate by BL [63,64]. These findings indicated that the negative feedback regulation of BR biosynthetic genes was common in both dicot and monocot plants.

The BR signaling pathway was considered to be intact in the BR-deficient mutants. In view of the increased expression levels of *LTBSG1* and other BR biosynthetic genes in the BR non-treated mutant *ltbsg1* than those in WT, the feedback regulation of BR biosynthetic genes might be regulated through the BR signaling pathway. In addition, the expression result of BR signaling genes revealed the altered expressions between mutant and WT (Figure 8E), which indicated that their responses to the defect of *LTBSG1* was changed. Thus, they were required for feedback regulation of BR biosynthesis genes. *BRI1* encoded a receptor kinase and could perceive the BR signal on cell surface [33]. *BRI1* was higher expressed in mutant *ltbsg1* compared with WT, we proposed that, thus, the highly sensitivity of mutant *ltbsg1* to 24-EBL might be caused by the accumulation of the BR receptor kinase. This similar result was also observed in the BR-deficient mutants *d2* and *d11*. In the study of *Arabidopsis*, the BZR1 protein, a transcription factor in BR signaling pathway, could mediate the feedback inhibition of the BR biosynthetic genes such as *BRD1*, *CPD* and *DWF4* [34]. The significantly higher expression of *BZR1* in mutant *ltbsg1* also confirming its role in BR response. Therefore, we proposed that this negative feedback regulation of the BR biosynthetic genes was regulated through BR-signaling molecules, such as the BRI1 and BZR1 proteins. The pathway of BR signaling introduction was a complicated network regulation, which involved in many kinds of receptor kinases, a series of phosphorylation and dephosphorylation reactions and transcription factor transmitting the signal to the target genes downstream [65,66]. Therefore, it needs more studies and efforts to uncover the roles of these genes they worked in the BR pathways.

In addition, the knock-out lines of *LTBSG1* displayed typical development defects of BR related mutants such as *ltbsg1*, *brd1*, *d2* and *d11*, which could also be restored by 24-EBL. Moreover, the top branch elongation and shorter grains were clearly identified in the knock-out plants (Figure 9). This work further confirmed the important role of *LTBSG1* in regulating panicle and grain development by BR biosynthesis pathway.

In this study, besides the new phenotype of longer top branch and shorter grain, the mutant showed the pleiotropic phenotypes such as dwarfism, erect lamina joint, poor fertility and late maturity, which was consistent with the phenotypes of BR related mutants [17,40]. This indicated that the BR biosynthesis pathway or the BR hormone itself played important roles in plant various development processes. To gain insight into the function of *LTBSG1*, the gene ontology enrichment analysis of differentially expressed genes (DEGs) in biological process was performed. The resulted revealed many DEGs involved in the different biosynthetic process, metabolic process and catabolic process. Except that, there were also some DEGs which were enriched in the process of biotic and abiotic stresses responses such as defense response to bacterium, fungus and oxidative stress (Table S4). Therefore, these results further confirmed the multiple roles of *LTBSG1*. The mechanism of plant growth development regulated by BR is a complicated process, which requires more research to clarify in the future.

## 5. Conclusions

In conclusion, *LTBSG1* was a new allele of *BRD2*, which was responsible for the longer top branch and shorter grain by involving in the brassinosteroid biosynthetic pathway.

## Figures and Tables

**Table 1:** The reaction monitoring conditions for protonated brassinolide ([M + H]^+^).

Analyte	Polarity	Precursor Ion (*m*/*z*)	Product Ion (*m*/*z*)	Cone Voltage (V)	Collision Energy (V)
brassinolide	+	481.6	445.3/315.3	48	29/57

*m*: The detected ion mass. *z*: The charge on the detected ion. *m*/*z*: The mass-to-charge ratio.

**Table 2:** Agronomic traits of WT and mutant *ltbsg1*.

Traits	WT	*ltbsg1*
Plant height (cm)	82.90 ± 1.30	54.41 ± 1.37 **
Top branch length (cm)	3.55 ± 0.21	5.63 ± 0.19 **
Primary branch length (cm) ^1^	7.60 ± 0.46	5.80 ± 0.35 **
Secondary branch length (cm)	2.13 ± 0.16	1.60 ± 0.21 **
No. of primary branches	12.67 ± 0.82	7.50 ± 1.05 **
No. of secondary branches	20.83 ± 1.17	1.83 ± 0.75 **
Grain length (mm)	8.12 ± 0.03	5.93 ± 0.02 **
Grain width (mm)	3.17 ± 0.02	2.83 ± 0.01 *
1000-grain weight (g)	25.83 ± 0.12	16.63 ± 0.03 **
No. of grains per panicle	190.90 ± 3.51	84.70 ± 3.83 **
Seed setting rate (%)	90.95 ± 1.38	8.42 ± 1.10 **

^1^ The primary branch length represented the average length of all primary branches without the top branch. Values represent the means ± SD (*n* = 10). * *p* < 0.05; ** *p* < 0.01.

## Supplementary Materials

The following are available online at http://www.mdpi.com/2073-4425/9/6/292/s1, Figure S1: Analysis of internode pattern and lamina joints of WT and *ltbsg1*, Figure S2: The detection of pollen vitality between WT and *ltbsg1*, Figure S3: Phylogenetic tree of LTBSG1 with 20 homologous proteins in different plants, Figure S4: The confirmation of mutation sites of *LTBSG1* three knock-out lines by sequencing, Table S1: Primer list of this study, Table S2: Predicated function analysis of candidate genes of *ltbsg1*, Table S3: Agronomic traits of Nipponbare (Nip) and three knock-out lines, Table S4: Gene ontology enrichment analysis for part of differentially expressed genes involved in biological process.
