# Supplementary material for: LTBSG1, a New Allele of BRD2, Regulates Panicle and Grain Development in Rice by Brassinosteroid Biosynthetic Pathway
Source: Genes (Basel). 2018 Jun 11;9(6):292. doi: 10.3390/genes9060292 (PMC6027417; doi:10.3390/genes9060292)
Supplement: Supplementary file 1 [file genes-09-00292-s001.pdf]

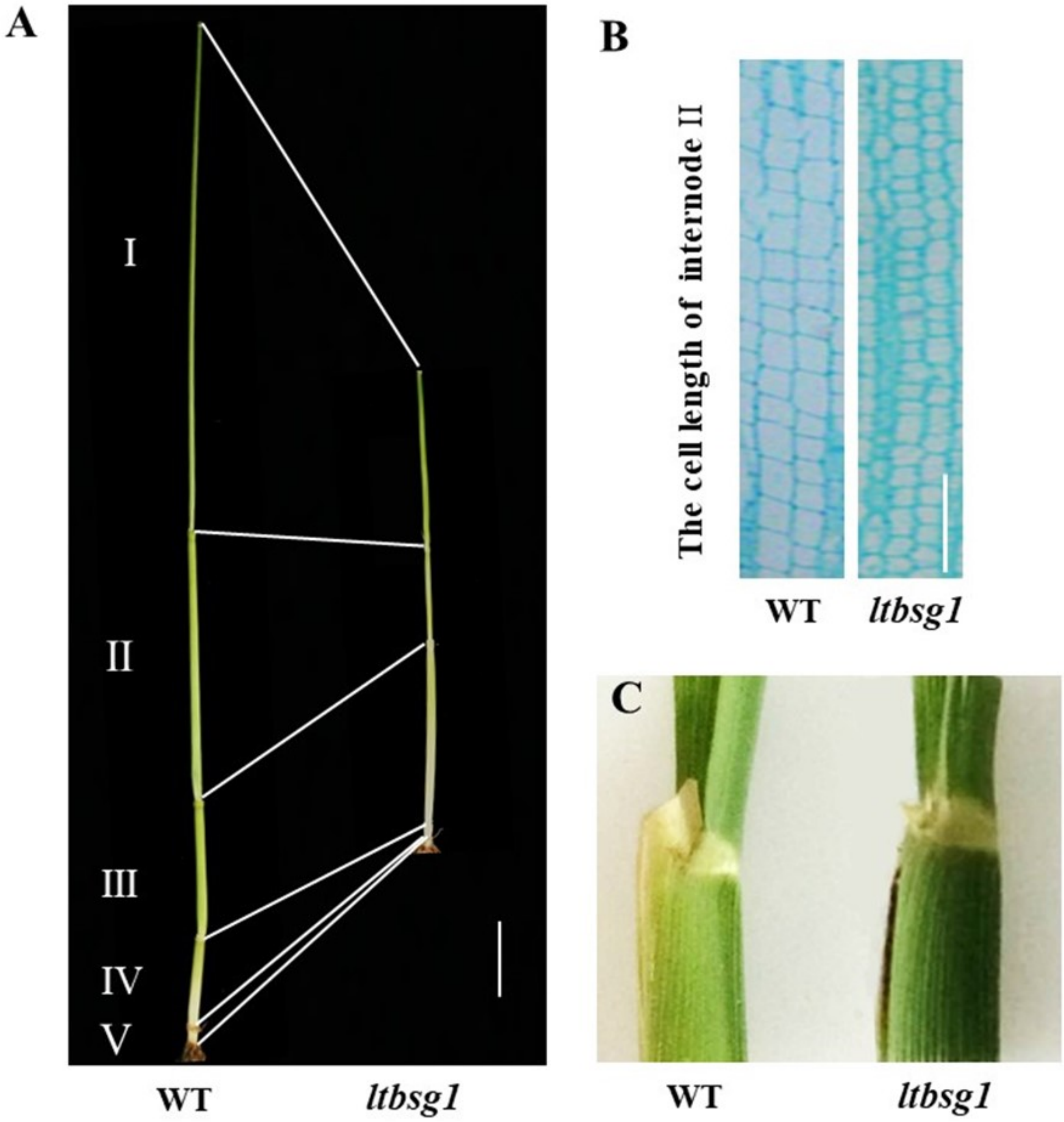

Figure S1. Analysis of internode pattern and lamina joints of WT and *ltbsg1*. (A) The internode pattern of WT and *ltbsg1*. Bar = 5 cm. (B) Longitudinal sections of the internode II in WT and *ltbsg1*. Bar = 100 μm. (C) The lamina joints of WT and *ltbsg1*.

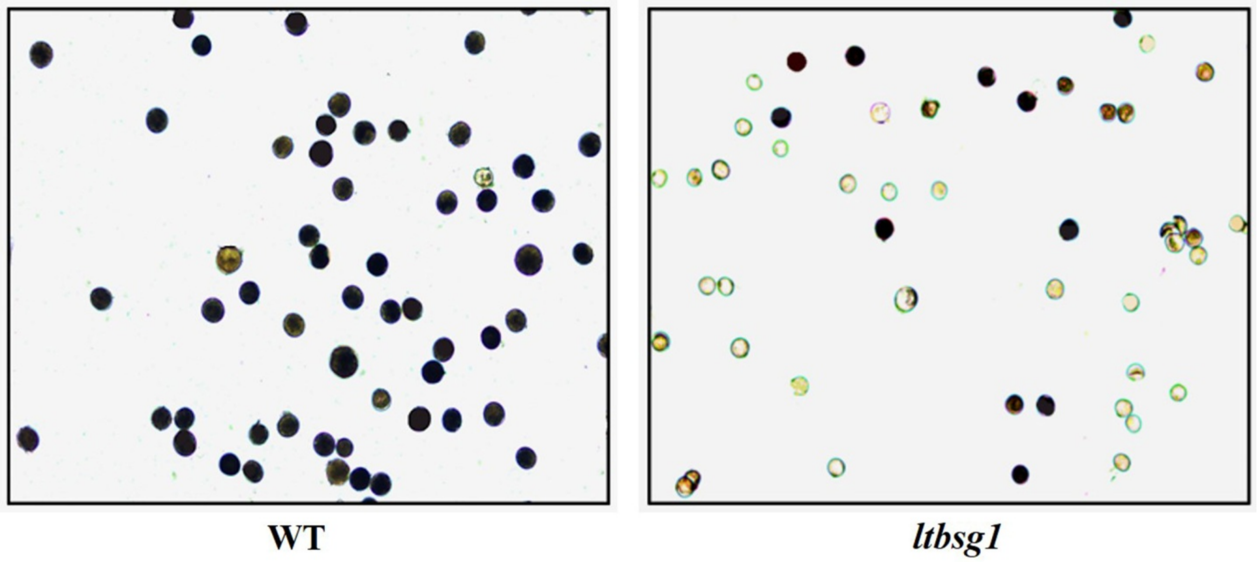

Figure S2. The detection of pollen vitality between WT and *ltbsg1*.

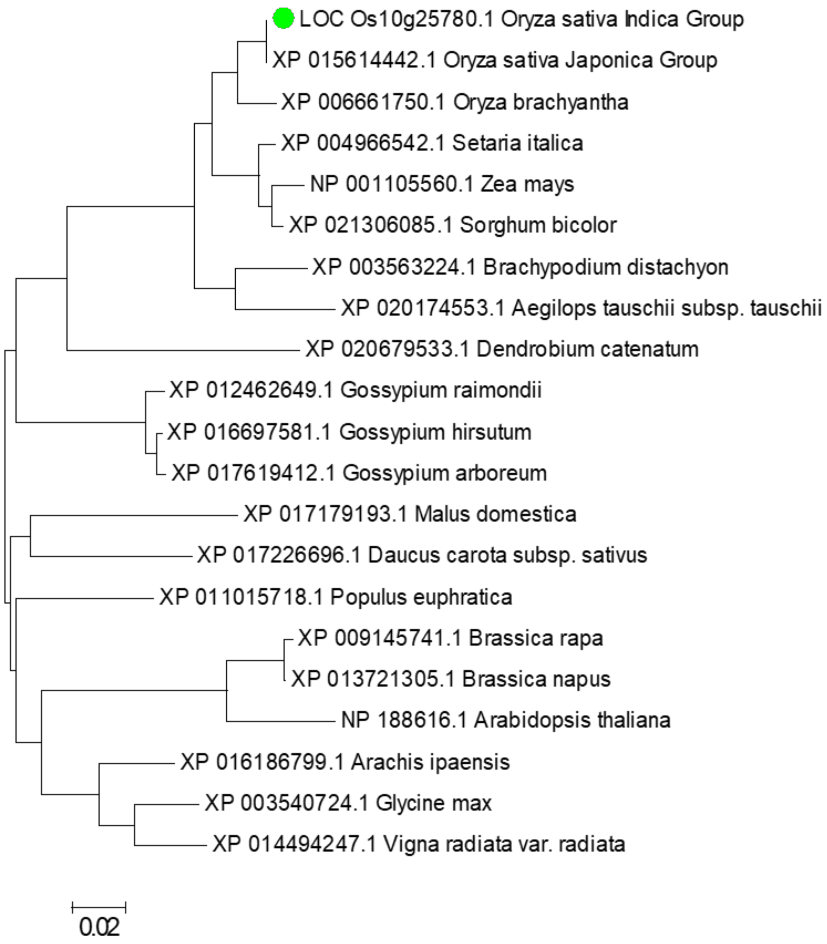

Figure S3. Phylogenetic tree of LTBSG1 with 20 homologous proteins in different plants. All the protein sequences were downloaded from the database of NCBI. The phylogenetic tree was constructed by MEGA5.10 program with the neighbor-joining method by 1,000 bootstrap replicates.

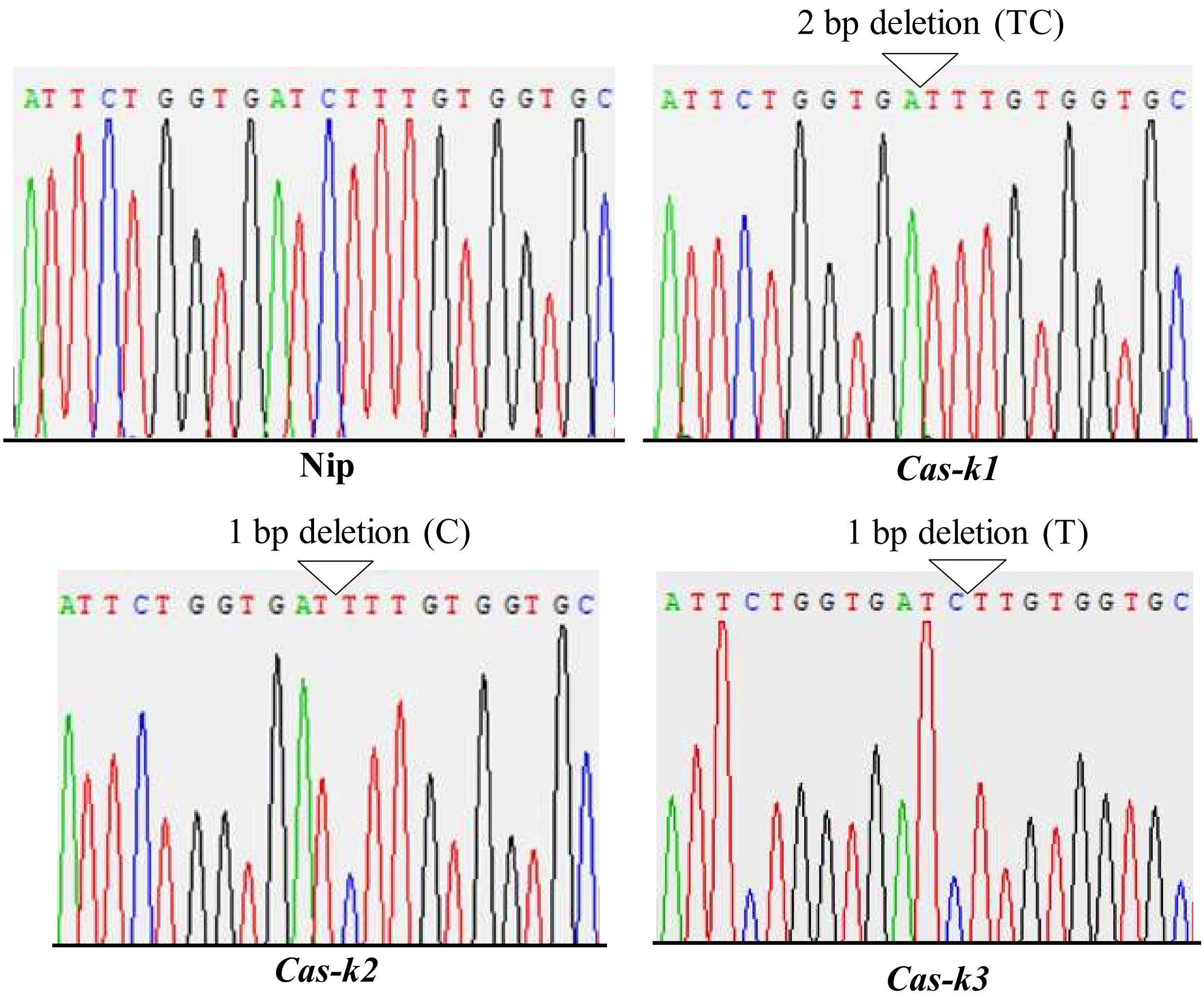

Figure S4. The confirmation of mutation sites of LTBSG1 knock-out lines by sequencing. The nucleotide deletions in Cas-k1, Cas-k2 and Cas-k3 were occurred in 173 th bp, 174 th bp and 175th bp, respectively.

Table S1. Primer list of this study.

(A) Primers for gene mapping

Marker	Forward primer (5′→3′)	Reverse primer (5′→3′)
RM596	ATCTACACGGACGAATTGCC	AGAAGCTTCAGCCTCTGCAG
z10-4	AACGTACACTCATCTTCCCTATTG	CTTCGAGATCTGGCTTTGTTTT
z10-5	AAGGATTCTGTGCAAGTTAGTTTT	AGAGAGGAAGCGGAGGAGAAT
z10-7	TGCAAATAATCCCAATCATAACTC	TTGCTAATGGATATGTGCTACCC
z10-9	AAGGCCATTATTTAAGCATCGTA	TAGGGTTCCATCACATAATAAAT
z10-10	GGTCTGATCGCCGCTTAGT	TTGTTGGTTTTTGTTTCAGATTAG
z10-11	GGCGCCGATTTGGAGGTA	TTGTTTGCTGGTAGTTCTCGTTCA
z10-12	GTTTCTATGGACCCGATACTGC	ACGAGCTTACCGCTGCTTT

(B) Primers for vector construction

Purpose	Forward primer (5′→3′)	Reverse primer (5′→3′)
Knock-out by CRISPR/Cas9	tgccgGTTGAGCCTCTTGTCAACATgt tttagagctagaaat	AAAACATGTTGACAAGAGGCTCAACgg cagccaagccagca
GUS assay	CCATGATTACgaattcATGGCGAAAG GAGTGGGTGAC	CTCAGATCTAccatggCTCCGCTGTATTGA CACAGTTACA
Subcellular localization	CGGGGGACGAGCTCggtaccATGGT AAAATGGAAGTGTGATGGATG	CAGTGAAAAGTTCTTCTCCTTTACTCAT tctagaCGCCTCATCAGCGTAGGC

(C) Primers for qRT-PCR

Gene	Forward primer (5′→3′)	Reverse primer (5′→3′)
OsActin	GTGGTCGCCCCTCCTGAAAG	GGCTTAGCATTCTTGGGTCCG
LTBSG1 /BRD2	AATCATAGCCGGACTGCAGCCAT	GCAGACCAGACATCGCCCAAATA
LP	GACAGTGAGGATAGGAGCCG	CGCCATCCAATCAATAGTTC
DEP1	TCAACATCTTCAAATGCTCCTGC	ATGGGTTACGACACCGTGGG
FZP	CACATTGGCTCGTACGGTCA	AAGAGGAAGTCGTGGCCGT
LAX2	CCGACGACGACCACCACCGGATT	TGGCGAGGCGGAGCCAGTCCCTG
TAW1	GCTGGAGAAGACGAAGAAAGATAG	CATTCCCCCTCCTCCTCC
GW2	CAGCAGCGCATTCCCAGTTTTC	GTGGTCAGCCGAGCACTCTC
qGW8	AGGAGTTTGATGAGGCCAAG	GCGTGTAGTATGGGCTCTCC
GL3.1	TCACAACTCCCAGGATAGG	TTTGTCTCGCTCGCTCAT
GS5	TTTGGCTGAGTATGCCTGGAG	ATTTGCGAAGAATGCACGAT
qSW5	GGGAGGGAGCAAGCGGAGGA	CTCGCCAAGTTGCCGGCTGC
SMG1	GTCATGATGAATTCCGATGGG	ACTCCTAGATCCCACGTCTCAA
TGW6	ACAGCCACAACGAGAATGTTCAAGA	TGTACGTGTAGGTGCTCCAGCCA
BRI1	CAGCTACTTGGCTATCTTGAA	CCATTCTTGTTGAAGGTGTACT
BU1	GTAGCCAGCTTGATCTCATCTC	GGGACGACTCTACTGCATCA
DLT	TCAATCCATTGCAGGGACGAT	ACGGCGACTTGCTGTACTCC
BAK1	CACCCACAGAAAGGTTGCTT	CATCATTGGCTAGACGAGCA
GSK2	TCGGTATCGTCTTTCAGGCTA	AAGCCCCCTAAATAACTGATACA
MDP1	TTATTGACCGGTACAACTCGCA	TCCAGTCCATCGATCTCATCC
BZR1	CCATCGCCGCCAAGATCTTCA	TGCCATCGCCGCCAAGATCTT
BSK3	CTACAGTACCAATCTGGCGTTTA	CAGGCATCTTGAAGCTAATCG
BLE2	GCTAGTTAGCTTACATGATGGC	GCGGGTGAACATCCTCGT
BRD1	GAGAAGAACATGGAATCACATCC	TCAGTAATCTTGAACGCGGATAT
BRD2	AATCATAGCCGGACTGCAGCCAT	GCAGACCAGACATCGCCCAAATA
D2	ATGTGATAACAGAGACGCTGCGGT	TGGTGACCAAGTGGTGAAGGAAGA
D11	AGTGAAGAGGGAGCATGAAGGCAT	ATCTGCAGGGCTGAAATTGTTGGG
CPD1	TCTTCTCCATCCCCTTTCCT	CACCCTCCGCCTCAAGA

Table S2. Predicated function analysis of candidate genes of *ltbsg1*

Accession number	Gene function annotation
LOC_Os10g25740	Cellulose synthase, putative, expressed
LOC_Os10g25750	Transposon protein, putative, CACTA, En/Spm sub-class
LOC_Os10g25760	Transposon protein, putative, CACTA, En/Spm sub-class, expressed
LOC_Os10g25770	Transcription initiation factor IIE subunit beta, putative, expressed
LOC_Os10g25780	FAD-linked oxidoreductase protein, putative, expressed
LOC_Os10g25800	Expressed protein
LOC_Os10g25810	Expressed protein
LOC_Os10g25830	Mitochondrial carrier protein, putative, expressed
LOC_Os10g25850	Nuclear transcription factor Y subunit, putative, expressed
LOC_Os10g25870	Dirigent, putative, expressed
LOC_Os10g25880	Retrotransposon protein, putative, unclassified, expressed
LOC_Os10g25890	Retrotransposon protein, putative, unclassified, expressed
LOC_Os10g25900	Dirigent, putative, expressed

Table S3. Agronomic traits of Nip and three knock-out lines

Traits	Nip	*Cas-k1*	*Cas-k2*	*Cas-k3*
Plant height (cm)	76.38 ± 2.85	31.55 ± 2.07**	30.44 ± 2.86**	32.74 ± 3.15**
Top branch length (cm) [1]	3.34 ± 0.31	4.46 ± 0.19**	4.09 ± 0.98**	5.22 ± 1.04**
Grain length (mm)	6.03 ± 0.05	4.41 ± 0.02**	4.43 ± 0.03**	4.36 ± 0.03**
Grain width (mm)	3.10 ± 0.01	2.14 ± 0.01**	2.10 ± 0.01**	2.24 ± 0.02**
No. of grains per panicle	58.37 ± 5.12	10.66 ± 2.62**	10.43 ± 1.54**	9.67 ± 2.17**
Seed setting rate (%)	88.28 ± 1.75	20.53 ± 2.18**	25.22 ± 2.53**	28.10 ± 3.12**

[1] The primary branch length represented the average length of all primary branches without the top branch. Values represent the means ± SD (n = 10). **, $P \leq 0.01$.

Table S4. Gene ontology enrichment analysis for part of differentially expressed genes involved in biological process

GO ID	GO term	Count in selection (out of 521)	Count in total genome (out of 10488)	Corrected *P* value
GO:0009889	Regulation of biosynthetic process	95	1069	1.52E-06
GO:2000112	Regulation of cellular macromolecule biosynthetic process	95	1069	1.52E-06
GO:008009	Regulation of primary metabolic process	95	1095	2.68E-06
GO:000604	Amino sugar metabolic process	9	17	2.68E-06
GO:0031323	Regulation of cellular metabolic process	95	1100	2.90E-06
GO:0016998	Cell wall macromolecule catabolic process	9	18	4.68E-06
GO:0009607	Response to biotic stimulus	7	13	5.75E-05
GO:1901136	Carbohydrate derivative catabolic process	9	26	0.00015
GO:1901565	Organonitrogen compound catabolic process	10	37	0.00049
GO:0006950	Response to stress	46	529	0.00630
GO:0006979	Response to oxidative stress	19	174	0.03893

Sequencing and analysis of transcriptome of the samples were performed in by Illumina Hiseq XTen platform. After QC and mapping of reads to genome of Oryza sativa.v7.0，Salmon (0.8.2) was used to calculate reads count and TPM (Transcripts Per Million) of unigene. Differentially expressed genes was calculate based on reads count of each gene using package DEseq2. Significant differentially expressed genes were picked with the following criteria: corrected *P* value <0.05 and *foldchange value* >2. The topGO (v 2.24.0) was used for GO enrichment analysis.
